# Thrombolysis for acute ischaemic stroke: development and update

**DOI:** 10.1093/braincomms/fcaf164

**Published:** 2025-04-28

**Authors:** Jiashuo Lin, Wenbo Zuo, Huijuan Jin, Quanwei He, Shengcai Chen, Bo Hu, Yan Wan

**Affiliations:** Department of Neurology, Union Hospital, Tongji Medical College, Huazhong University of Science and Technology, Wuhan 430022, China; Department of Neurology, Union Hospital, Tongji Medical College, Huazhong University of Science and Technology, Wuhan 430022, China; Department of Neurology, Union Hospital, Tongji Medical College, Huazhong University of Science and Technology, Wuhan 430022, China; Department of Neurology, Union Hospital, Tongji Medical College, Huazhong University of Science and Technology, Wuhan 430022, China; Department of Neurology, Union Hospital, Tongji Medical College, Huazhong University of Science and Technology, Wuhan 430022, China; Department of Neurology, Union Hospital, Tongji Medical College, Huazhong University of Science and Technology, Wuhan 430022, China; Department of Neurology, Union Hospital, Tongji Medical College, Huazhong University of Science and Technology, Wuhan 430022, China

**Keywords:** thrombolytics, acute ischaemic stroke, development, efficacy, safety

## Abstract

Thrombolytic therapy is a cornerstone in managing acute ischaemic stroke, marking significant advancements in treatment. Various generations of thrombolytics play crucial roles in different strategies, including intravenous thrombolysis, bridging therapy and thrombolysis beyond the conventional time window. The continuous development of thrombolytics has brought notable improvements. Compared to first-generation urokinase, second-generation alteplase and third-generation tenecteplase offer significant pharmacological advantages, such as enhanced fibrin specificity and longer half-lives. Tenecteplase demonstrates non-inferiority to alteplase regarding efficacy and safety, with the added benefit of a more convenient administration method. Ongoing trials continue to reveal additional evidence. Furthermore, other thrombolytic agents, including reteplase and non-immunogenic recombinant staphylokinase, are gaining increasing interest in the medical community. This review examines the structural characteristics, pharmacological properties, efficacy and safety profiles of these thrombolytic drugs. It also provides a detailed analysis of the performance of thrombolytic therapy in different acute ischaemic stroke patient subgroups, aiming to trace the evolution of these treatments and compare their effectiveness in acute ischaemic stroke. The goal is to offer a scientific basis for clinical practices and future development of thrombolytic therapies.

## Teaser

Lin *et al*. report that thrombolytic therapy remains pivotal in acute ischaemic stroke management, with tenecteplase emerging as a superior alternative to alteplase. Their review evaluates thrombolytic agents, strategies and advancements, providing comprehensive insights to guide clinical practice and future research in optimizing stroke treatment outcomes.

## Graphical Abstract

**Graphical Abstract:**
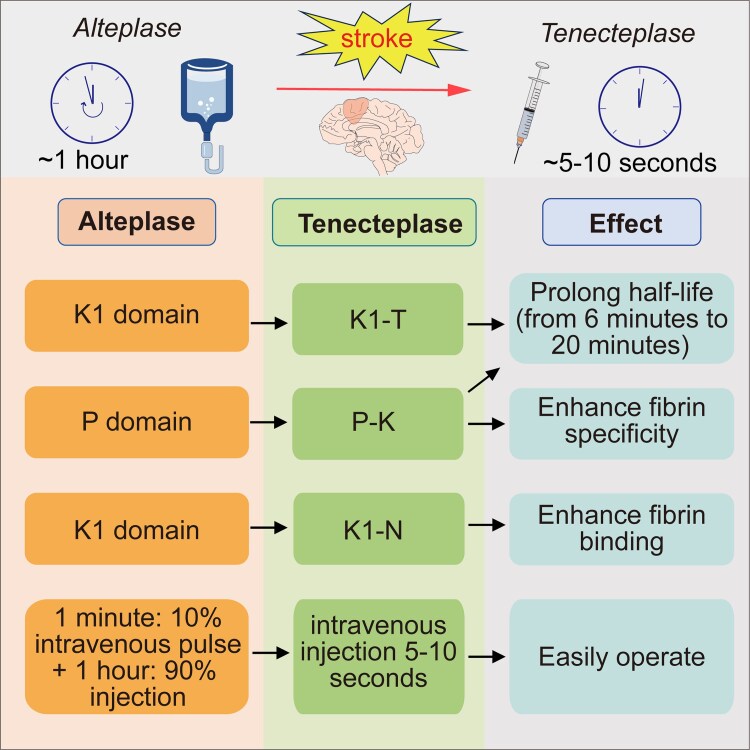

## Introduction

Stroke remains a major contributor to the global disease burden, consistently ranking among the leading causes of morbidity from 2010 to 2021.^1^ Ischaemic stroke, responsible for 60–70% of all strokes, is predominantly caused by acute arterial occlusion.^2^ Acute ischaemic stroke poses a severe health threat, causing rapid brain damage and neuronal injury after the ischaemic episode.^3^ Delayed treatment can result in permanent neurological deficits, underscoring the urgency for timely intervention in acute ischaemic stroke management.

Thrombolysis is a pivotal therapeutic approach in acute ischaemic stroke management, utilizing thrombolytic agents to activate the body’s fibrinolytic system, thereby facilitating clot dissolution and rapid reperfusion to salvage ischaemic brain tissue. The introduction and implementation of recombinant tissue plasminogen activator (rt-PA) marked a significant milestone in recanalization treatment for acute ischaemic stroke, significantly improving functional outcomes in patients.^4^ Due to its superior effectiveness and safety profile, rt-PA has become the most widely used thrombolytic agent globally.

The newer drug, tenecteplase (TNK-tPA), has undergone rigorous international clinical trials, demonstrating efficacy and safety comparable to rt-PA. In addition, with a more convenient administration method, the latest guidelines have assigned TNK-tPA an equivalent recommendation to rt-PA in intravenous thrombolysis.^5^ Although TNK-tPA has been recommended for use in bridging therapy,^5-7^ ongoing studies such as BRIDGE-TNK, DIRECT-TNK and TRACE-V are expected to yield new evidence and perspectives in the future. The release of the RAISE study has further propelled reteplase (r-PA) into the spotlight, generating intense research interest.^8^ The gradual development of related studies is anticipated to provide more treatment strategies for thrombolytic therapy in acute ischaemic stroke.

The integration of these novel agents, along with the continuous refinement of thrombolytic therapy, underscores the evolving landscape of acute ischaemic stroke management. This evolution offers hope for improved outcomes and more personalized treatment options for acute ischaemic stroke patients. As the repertoire of thrombolytic agents expands, with each generation witnessing incremental improvements, it is crucial to examine both the historical progression and current advancements of these drugs for acute ischaemic stroke thrombolysis. This review covers historical milestones from the initial use of streptokinase and urokinase, to the widespread adoption of rt-PA, and the emergence of TNK-tPA, r-PA and other new drugs. By analysing the structural characteristics, pharmacological properties, efficacy and safety profiles of these agents, and evaluating the performance of thrombolytic therapy across different subgroups of acute ischaemic stroke patients, we can trace their evolution and compare their effectiveness in acute ischaemic stroke management. This analysis aims to provide a scientific basis for clinical practice and the future development of thrombolytic drugs.

## Materials and methods

The PubMed, Web of Science and Google Scholar databases were retrieved in detail, and we used the following keywords for the retrieval: ‘thrombolytic drugs’, ‘thrombolysis’, ‘acute ischaemic stroke’, ‘tenecteplase’. ‘alteplase’, ‘streptokinase’, ‘urokinase’, ‘pharmacology’, ‘structure’, ‘outcome’ and ‘safety,’ etc., and selected some of pertinent articles for citation.

## Development of thrombolytics

Streptokinase and urokinase were the first substances discovered and validated for their fibrinolytic properties. However, three large studies assessing streptokinase’s efficacy and safety in acute ischaemic stroke were halted due to safety concerns, particularly high rates of intracerebral haemorrhage and mortality.^9-11^ Thus, streptokinase was not approved for acute ischaemic stroke. Additionally, a clinical trial on recombinant urokinase precursor (r-proUK) showed significant improvement in 90-day clinical outcomes but also an increased risk of intracranial bleeding.^12^ Urokinase was not widely used for acute ischaemic stroke globally, apart from in China, especially in remote areas where better thrombolytic drugs cannot be obtained. The ongoing quest for better thrombolytic agents aims to achieve faster reperfusion, higher specificity for fibrin to minimize bleeding complications, prolonged circulation for reduced dosing, improved resistance to inhibitors and lower antigenicity.^13^

The second-generation agents, such as tissue plasminogen activator (t-PA), addressed some issues associated with first-generation drugs, particularly systemic bleeding from plasminogen activation. The specificity for fibrin of t-PA is a key advantage,^14^ significantly enhancing its activity in the presence of fibrin. This specificity allows t-PA to form a complex with plasminogen and fibrin, resulting in increased affinity for plasminogen.^15^ Consequently, t-PA has been widely recommended for use in acute ischaemic stroke worldwide.

The third-generation agents, mainly variants of t-PA such as TNK-tPA and r-PA, exhibit improved stability, safety and efficacy. These agents have longer half-lives, enhanced fibrin specificity, better thrombus penetration and increased resistance to inhibitors.^16^ To date, TNK-tPA has been approved for acute ischaemic stroke thrombolysis in China, the American Stroke Association (ASA) and European Stroke Organisation (ESO), due to its good efficacy and safety profile demonstrated in trials such as AcT and TRACE-2.^17,18^ Mounting ongoing clinical researches are expected to provide more evidence for the application of TNK-tPA. In 2020, non-immunogenic recombinant staphylokinase was registered in Russia for acute ischaemic stroke treatment, with studies suggesting non-inferiority to rt-PA in terms of efficacy and safety, though further research is needed.^19^ The recently released RAISE trial conducted in China found that r-PA was not inferior to rt-PA in efficacy for acute ischaemic stroke treatment,^8^ offering clinicians and patients economic, convenient and diverse thrombolytic drug options.

## Structure, pharmacological action and metabolism of thrombolytics

Comprehending the structural characteristics, mechanisms of action and metabolic pathways of various thrombolytic agents is essential, as these factors directly influence their clinical performance and provide critical insights into the molecular-level evolution and optimization of these drugs (see Table 1).

**Table 1:** Structure, pharmacological action and metabolism of thrombolytics

Generation	Thrombolytic	Structure		Domain function	Pharmacological characteristics	Mode of administration
The first generation	Streptokinase	α		Formation of plasminogen active conformation in the complex Substrate identification	Indirect action Non-fibrin-specific	Not approved
		β		High-affinity binding in the activation of plasminogen ‘chaperones’ Promotes the binding and processing of plasminogen ‘substrates’		
		γ		Proteolysis		
	Urokinase	EGF		Receptor binding	Direct action Non-fibrin-specific	1 000 000∼1 500 000 IU, dissolved in 100–200 ml of normal saline, intravenous infusion for 30 min（approved in China）
		K		Provides the ability to bind to fibrin		
		P		Catalytic activity		
The second generation	Alteplase	F		High affinity for fibrin	Direct action High fibrin specificity Short half-life	0.9 mg/kg (maximum dose of 90 mg) was given intravenously, of which 10% of the dose was given intravenously in the first 1 min, and the remaining 90% was continuously instilled for 1 h
		EGF		Binding to EGF receptor binding		
		K1		Binding to mannose receptors expressed in hepatic endothelial cells and Kupffer cells		
		K2		Proteolysis; Lower affinity for fibrin		
		serine protease P		Catalytic activity Binding to plasminogen activator inhibitor-1		
		Carbohydrates		Plasma clearance		
The third generation	Tenecteplase	F		High affinity for fibrin	Direct action High specificity and affinity for fibrin Extended half-life Increased resistance to PAI	0.25 mg/kg intravenous bolus for 5–10 s
		EGF		Binding to EGF receptors		
		K1	T103N	Decreases clearance and prolongs half-life		
			N117Q	Improves the mutant’s fibrin binding capacity		
		K2		Proteolysis Lower affinity for fibrin		
		P	KHRR (296–299) AAAA	The P structure is catalytically active The mutation significantly improved fibrin specificity and drug resistance to plasminogen activator inhibitor-1		
	Reteplase	K2		Specifically binding to fibrin, but with low affinity	Direct action Fibrin specificity is retained with low affinity Extended half-life	Two intravenous injections, 18 mg each time, 2 min, 30 min between the two doses (in the test)
		P		Catalytic activity		
Other	Non-immunogenic recombinant staphylokinase	α（with β-grasp folds） with mutation		Activation of plasmin High resistance to α2 antiplasmin There is no immunogenicity after mutation	Indirect action High specificity for fibrin	A single intravenous bolus of 15 mg, regardless of the patient’s weight (Russia)

EGF, Epidermal growth factor (domain); F, Fibrin binding finger domain; K, Kringle domain.

### The first generation of thrombolytics: streptokinase and urokinase

Streptokinase, an indirect activator of plasminogen, is a 47 kDa protein composed of 415 amino acids, divided into three domains: α, β and γ.^20^ The α-domain primarily mediates the formation of the active conformation of plasminogen in the streptokinase–plasminogen complex, the β-domain is responsible for high-affinity binding during plasminogen ‘partner’ activation, and the γ-domain is associated with the stability of the streptokinase-type plasminogen complex and induces its proteolytic activity. Streptokinase exerts its thrombolytic effect by forming a complex with plasminogen in a stoichiometric ratio.^21-23^

Urokinase is a 54 kDa protein with 411 amino acids^24^ and contains the epidermal growth factor (EGF) domain, a K domain (a flexible linker) and a serine protease domain P.^21,25,26^ This structure enables urokinase to directly convert plasminogen to plasmin by cleaving peptide bonds. Both streptokinase and urokinase are primarily metabolized in the liver, with half-lives ranging from 14 to 20 min for streptokinase and 18–23 min for urokinase.^27^ However, due to their lack of specificity for fibrin, both drugs can lead to systemic activation of plasminogen, increasing the risk of bleeding. This limitation prompted the development of second-generation thrombolytic drugs with higher specificity for fibrin.

### The second generation of thrombolytics: rt-PA

The second-generation thrombolytic agent, t-PA, is a 527-amino acid glycoprotein with five domains: F (fibronectin-like finger), EGF, two K (Kringle) and P (serine protease) domains. The F domain is with high affinity for fibrin, EGF and K1 domains are for receptor binding, K2 domain plays a role in proteolysis and fibrin binding, and P domain is in charge of catalytic activity and interaction with PAI-1.^21,28-30^ The modified form of t-PA, rt-PA, has a lower affinity for free plasminogen and fibrinogen compared to urokinase but binds tightly to fibrin,^25^ which enhances rt-PA’s activity and its affinity for plasminogen.^15^ However, rt-PA can still cause the decomposition of plasminogen and fibrinogen, and it has a very short half-life of only 6 min.^31^ Additionally, it is rapidly inactivated by PAI-1 (the main determinant of thrombolysis resistance),^32^ necessitating continuous intravenous infusion and large doses for clinical application.^28^

### The third generation of thrombolytics: represented by TNK-tPA

The represented third generation of thrombolytic, TNK-tPA, derived from t-PA, incorporates mutations at specific sites to alter its pharmacological properties. The T103N mutation significantly reduces drug clearance and prolongs the plasma half-life. The N117Q mutation improves fibrin binding, and the KHRR (296–299) AAAA mutation significantly increases fibrin specificity and resistance to PAI-1.^14,31^ These mutations make TNK-tPA a potentially more advantageous thrombolytic drug, with a longer plasma half-life and improved thrombolytic effect.

Additionally, r-PA, a simplified version of t-PA, consists only of the Kringle 2 and serine protease domains, omitting other domains. This structure reduces the drug’s confinement to the thrombus surface, enhancing its ability to penetrate and improve fibrinolytic activity. The absence of the K1 and EGF domains reduces receptor binding and metabolic clearance, prolonging the half-life and reducing the dosage required.^29^

The third one, staphylokinase, a 15.5 kDa protein composed of 136 amino acids, forms a single-domain structure through multiple β-sheet folds.^33,34^ It exhibits an indirect activation mechanism by forming a 1:1 complex with plasmin, modifying plasmin’s broad substrate specificity to selectively cleave the Arg561-Val562 bond within another plasminogen molecule, generating active plasmin. Residues 26, 42–50, 65–69 and 75 are crucial for binding to plasmin’s ‘partner’, while residues 11–16, 46–50, 65–69 and 97–98 are involved in plasminogen’s ‘substrate’ interaction and processing. The first 10 N-terminal residues are dispensable for its activity and can be cleaved by active plasmin.^21^

## Clinical efficacy of thrombolytics across generations in the treatment of acute ischaemic stroke

With advances in acute ischaemic stroke research, the use of thrombolytic agents across generations has become more refined and diverse. The following section systematically compares their clinical efficacy, aiming to support the optimization of individualized treatment strategies.

### Streptokinase

Streptokinase was first found no significant differences in 6-month mortality and disability rates in stroke patients compared with the placebo in the MAST-I in Italy^11^ and the following MAST-E study also failed to demonstrate a benefit in primary efficacy endpoints between the streptokinase and placebo groups. In addition, safety concerns led to the recommendation against routine use of streptokinase for acute ischaemic stroke thrombolysis.^9^ With the time window advanced to 4 h after onset, the Australian Streptokinase (ASK) trial still failed to confirm its advantage, while the trial was halted prematurely due to high mortality and intracerebral haemorrhage rates in patients treated beyond 3 h post-stroke.^10^ Consequently, streptokinase was not approved for clinical use in acute ischaemic stroke.

### Urokinase

In contrast to streptokinase, urokinase is one of the few thrombolytic agents approved in China for treating acute ischaemic stroke with a 6-h therapeutic window, primarily serving as an alternative when rt-PA is unavailable. The MELT trial in Japan administered urokinase intra-arterially within 6 h of cerebral artery occlusion, using multiple criteria to assess clinical efficacy. Although the primary endpoint of favourable outcome (mRS 0–2) was not superior to the control group, the secondary endpoint of excellent functional outcome (mRS 0–1) was significantly higher in the urokinase group (42.1 versus 22.8%, *P* = 0.045).^35^ The PROACT and PROACT II trials demonstrated that r-proUK significantly improved clinical outcomes, albeit with an increased risk of bleeding.^12,36^

A multicentre retrospective study in China found patients treated with low-dose urokinase (1 000 000 IU) had a similar functional outcome but a lower risk of extracranial bleeding compared to those treated with high-dose urokinase (1 200 000–1 500 000 IU), suggesting smaller doses might be more beneficial.^37^ The subsequent INTRECIS trial in China, enrolling 3810 patients, demonstrated that intravenous urokinase was potentially as efficacious as rt-PA in treating acute ischaemic stroke of mild to moderate severity within 4.5 h of onset.^38^ These findings support the inclusion of urokinase thrombolysis in Chinese guidelines for stroke management, though it has not been approved by the FDA.^5^ And due to its affordable price, urokinase has also been used in adjuvant thrombolysis in intravascular thrombectomy for acute ischaemic stroke in China.

### Recombinant tissue plasminogen activator

#### Standard use of rt-PA

rt-PA is the first thrombolytic agent approved by the FDA for acute ischaemic stroke, with widespread global adoption and well-documented efficacy. The NINDS study, enrolling 624 acute ischaemic stroke patients, found that administering 0.9 mg/kg of rt-PA intravenously within 3 h post-incident significantly improved neurological function at 3 months compared to placebo.^4^ Patients treated with rt-PA were at least 30% more likely to have minimal or no disability after 3 months compared to placebo,^4^ supporting the FDA’s approval of rt-PA for acute ischaemic stroke in 1996^39^ and subsequent European endorsement.^40^

The thrombolysis window for rt-PA was further explored in the ATLANTIS trial, which investigated efficacy in patients presenting within 3–5 h of onset. No significant advantage at the 90-day endpoint was found, and an increased risk of symptomatic intracerebral haemorrhage was observed.^41^ The EPITHET trial (a Phase II trial) discovered that in patients with a mismatch of perfusion-weighted imaging and diffusion-weighted imaging (DWI) within 3–6 h of symptom onset, rt-PA did not mitigate infarct growth but was significantly associated with increased reperfusion, a potential therapeutic window extension beyond 3 h for certain patients.^42^ The ECASS III trial (a Phase III trial) conducted in Europe, reported that rt-PA use within 3–4.5 h significantly improved clinical outcomes, extending the thrombolysis window to 4.5 h worldwide.^43^

#### Extending the treatment window

Extending the rt-PA administration window remains an area of inquiry. A meta-analysis of studies, including ECASS, ATLANTIS, NINDS and EPITHET, suggests shorter treatment durations are optimal, as risks may outweigh benefits beyond 4.5 h.^44^ The international, multicentre study IST-3 trial demonstrated that rt-PA thrombolysis within 6 h improved 6-month functional outcomes, even in patients over 80 years old.^45^ A relevant meta-analysis concurs with this report, although the greatest benefit is observed within the first 3 h.^46^ The EXTEND study showed better primary outcomes (mRS 0–1) for rt-PA users compared to placebo in patients with a favourable perfusion-imaging profile between 4.5 and 9 h after stroke onset.^47^ Moreover, the WAKE-UP study indicated that intravenous rt-PA guided by MRI evaluation (DWI/FLAIR mismatch) in patients with uncertain onset times resulted in significantly better functional outcomes.^48,49^ A meta-analysis of EXTEND, ECASS4-EXTEND and EPITHET studies confirmed that rt-PA administration within 4.5–9 h, or in wake-up stroke patients, following CT perfusion or perfusion diffusion MRI indicating salvageable brain tissue, was associated with improved functional outcomes compared to placebo.^50^ Thus, rt-PA is recommended for patients beyond the standard thrombolysis window, relying on advanced neuroimaging to screen candidates.^6^

#### Bridging therapy with rt-PA

rt-PA plays a pivotal role in bridging therapy for acute ischaemic stroke. The THRACE trial demonstrated the superiority of combined rt-PA and mechanical thrombectomy over rt-PA alone (OR 1.55, 95% CI 1.05–2.30; *P* = 0.028), indicating functional benefits at 3 months.^51^ A meta-analysis supported the combination of intra-arterial thrombectomy with intravenous thrombolysis within the first 6–8 h of anterior circulation large vessel ischaemic stroke.^52^ The DIRECT-MT study in China found standalone mechanical thrombectomy was non-inferior compared to bridging therapy.^53^ However, the SKIP (Japan),^54^ MR CLEAN (Europe),^55^ DIRECT-SAFE (Australia, New Zealand, China and Vietnam)^56^ and SWIFT DIRECT (Europe and Canada)^57^ trials reported that standalone thrombectomy did not demonstrate non-inferiority compared to bridging therapy. A similar conclusion was validated in a pertinent meta-analysis.^58^ Thus, based on the available evidence, both Chinese and US guidelines recommend intravenous thrombolysis prior to mechanical thrombectomy for eligible acute ischaemic stroke patients.^7,59^ Additionally, the individualized decision and cost-effectiveness analysis should be further studied when considering patient characteristics, drug shortages, or delays in administration might counteract the benefits of intravenous thrombolysis prior to endovascular treatment.^58^

#### In intra-arterial thrombolysis

Intra-arterial thrombolysis offers an easier access of the drug and more effective thrombolytic activity within the microcirculation, not impeded by the coexistence of more proximal occlusions, which could further improve hypoperfusion and prognosis of acute ischaemic stroke patients. In the CHOICE trial, intra-arterial rt-PA following mechanical thrombectomy showed a potential increase in excellent neurological outcomes at 90 days compared to placebo.^60^ However, further research with larger sample sizes is needed. A systematic review at the 2023 International Stroke Conference concluded that intra-arterial thrombolysis as an adjunct to mechanical thrombectomy is safe, particularly for subgroups such as those under 65 years, with baseline NIHSS scores <10 or 10–19, those who received intravenous thrombolysis, those with post-procedural mTICI grade 3, and those with basilar artery occlusion.^61^ However, more evidence is needed to explore the efficacy of intra-arterial thrombolysis combined with mechanical thrombectomy.

#### In mobile stroke units

Intravenous thrombolysis also plays a critical role in pre-hospital emergency care. Mobile stroke units, specialized ambulances with experienced acute stroke care professionals, can diagnose and treat stroke patients on the scene, reducing thrombolysis times by an average of 25 min.^62^ The B_PROUD study in Berlin demonstrated that mobile stroke units shortened acute ischaemic stroke treatment time, increased thrombolysis rates, and enhanced pre-hospital triage, significantly associating mobile stroke unit deployment with lower overall disability rates at 3 months post-stroke.^63^ In this trial, mobile stroke unit-assigned patients experienced a median time of 50 min from dispatch to initiating thrombolysis, compared to 70 min for those without mobile stroke unit support. The *post hoc* analysis of the associations between onset-to-treatment time and outcomes among 687 patients treated with t-PA found that shortened thrombolysis times were associated with excellent outcomes (mRS 0–1). The BEST-MSU trial similarly revealed lower disability rates at 90 days for acute ischaemic stroke patients treated with mobile stroke units compared to standard emergency medical services care.^64^ The ESO guidelines emphasize mobile stroke unit treatment’s safety and recommend its use in pre-hospital assessment of suspected stroke patients.^65^

#### Use in special populations

##### Elderly patients

The elderly population constitutes a distinct subset of stroke patients, necessitating careful considerations and tailored treatment plans, including the use of intravenous thrombolysis. A systematic review and meta-analysis of cohort observational studies indicates that elderly patients aged 80 and above have higher mortality and less favourable clinical outcomes compared to those under 80,^66^ highlighting the need for larger trials comparing thrombolysis and non-thrombolysis treatments in this age group. In the IST-3 study, which included 1617 patients older than 80 years, it was found that rt-PA thrombolysis provided significant benefits.^45^ A systematic review published in *the Lancet* in 2014 conducted a meta-analysis of nine clinical trials involving 6756 acute ischaemic stroke patients treated with rt-PA, showing that people over 80 years old could still benefit from intravenous thrombolysis with rt-PA within 3 h of onset.^67^

In 2017, a study by the Karolinska Institute in Sweden compared treatment results and risks of intravenous thrombolysis within 3 h versus 3–4.5 h in patients over 80 years old.^68^ This study included 14 240 acute ischaemic stroke patients aged 80 years and above who received thrombolysis within 4.5 h (3558 cases within 3–4.5 h). It found no significant differences in outcomes between the groups and thrombolysis within 3–4.5 h still improved the prognosis, although the rate of symptomatic intracerebral haemorrhage in the 3–4.5 h group was higher. This suggests that elderly patients should not reject intravenous thrombolysis within the time window solely due to age when there are no contraindications. The ESO guidelines recommended intravenous thrombolysis with rt-PA for acute ischaemic stroke patients with onset time <4.5 h and age >80 years.^6^

In 2022, the large multicentre study (TRISP) collected prospective cohort data to compare the rate of symptomatic intracerebral haemorrhage, mortality and poor functional prognosis between patients aged ≥90 years and patients aged <90 years (defined as mRS 3–5 for patients with pre-stroke mRS ≤ 2; or mRS 4–5 for patients with pre-stroke mRS ≥ 3).^69^ The results showed no significant differences in the incidence of symptomatic intracerebral haemorrhage between the two groups. However, patients aged ≥90 had a higher risk of mortality and poor functional prognosis, and it is similar in the sample population of centenarians (*n* = 21). Therefore, for patients aged 90 and above, physicians should carefully consider the risk profile over the benefits.

##### Patients with renal insufficiency

In patients with renal insufficiency, the utilization of intravenous thrombolysis requires careful consideration due to the correlation between renal insufficiency and the safety and prognosis following thrombolysis. A *post hoc* analysis of the ENCHANTED study revealed that in acute ischaemic stroke patients who met eligibility criteria and underwent rt-PA treatment, renal insufficiency was associated with increased mortality but not with disability or symptomatic intracerebral haemorrhage.^70^ No significant difference in efficacy was observed between low-dose and standard-dose rt-PA in these patients. However, several studies have suggested that moderate to severe renal insufficiency following intravenous thrombolysis is linked to a heightened risk of cerebral haemorrhage or worsened prognosis. The severity of renal impairment must be considered when assessing the risk-benefit ratio of rt-PA.^71-74^ Low glomerular filtration rate may serve as a better indicator of symptomatic intracerebral haemorrhage risk than age.^73^ Another analysis indicated that the benefits of rt-PA were not different between stroke patients with and without chronic kidney disease at an unknown time of onset, but this applied only to mild to moderate or pre-dialysis chronic kidney disease.^75^ Current analyses do not provide definitive evidence for the efficacy of intravenous thrombolysis in stroke patients with chronic kidney disease who are awake or have an unknown onset time. Therefore, in patients with moderate to severe renal insufficiency, the application of intravenous thrombolysis should be cautiously weighed against potential complications and adverse outcomes, given the uncertain benefits.

##### Patients with atrial fibrillation

In patients with acute ischaemic stroke and atrial fibrillation, the decision to administer thrombolysis is similarly nuanced. The VISTA study compared the acute ischaemic stroke patients with or without a history of atrial fibrillation and found that the benefits of thrombolysis were similar in both atrial fibrillation and non-atrial fibrillation patients,^76^ with respective OR for improved outcomes of 1.44 and 1.53, after adjusting for baseline NIHSS and age. Atrial fibrillation was not independently associated with stroke outcomes, and compared to untreated patients, those who received thrombolytic therapy had a comparable advantage in outcomes, regardless of atrial fibrillation presence. These findings support the use of rt-PA in acute ischaemic stroke patients with atrial fibrillation. However, the IST-3 subgroup analysis did not reveal a significant difference between the rt-PA and control groups in the atrial fibrillation subgroup.^77^

Moreover, a study of registries of Lille (France) and Belgrade (Serbia) found that rt-PA had a worse prognosis for patients with atrial fibrillation receiving intravenous administration compared to those without atrial fibrillation, potentially due to their older age and more severe stroke upon admission.^78^ A meta-analysis suggests that atrial fibrillation is associated with adverse outcomes following thrombolysis.^79^ The controversy among conclusions indicates that the current evidence is inconclusive regarding intravenous thrombolysis for acute ischaemic stroke patients with atrial fibrillation, emphasizing the need for more data to determine the optimal management strategy in this population.

##### Patients with cerebral microbleeds

In the context of patients with cerebral microbleeds, a secondary analysis from the THAWS randomized clinical trial has suggested that ≥5 cerebral microbleeds might be linked to adverse outcomes following intravenous thrombolysis.^80^ A meta-analyses has further established that the presence of cerebral microbleeds confers an elevated risk of symptomatic intracerebral haemorrhage post intravenous thrombolysis (RR, 2.36; 95% CI, 1.21–4.61; *P* = 0.01).^81^ Moreover, a count of ≥10 cerebral microbleeds was correlated with a heightened propensity for symptomatic intracerebral haemorrhage. Conversely, two additional secondary analyses of the WAKE-UP trial data have determined that neither the existence of cerebral microbleeds nor white matter hyperintensities exerts a substantial influence on the safety profile or the functional results of intravenous thrombolysis.^82,83^ Consequently, in light of the current scientific evidence and treatment guidelines across different nations, the presence of fewer than 10 cerebral microbleeds should not be considered the solitary criterion for denying intravenous thrombolysis. Instead, the clinical scenario of such patients should be carefully evaluated on an individual basis, with a comprehensive assessment of the potential risks and benefits from intravenous thrombolysis to guide the decision-making process.

##### Patients with minor stroke

In acute ischaemic stroke, more than half of the patients present with minor strokes, while the application of intravenous thrombolysis in minor stroke is a debatable point.^84,85^ Two large-scale randomized controlled trials conducted in recent years, PRISMS and ARAMIS, indicated that intravenous rt-PA thrombolysis did not confer additional benefits over antiplatelet therapy in patients with minor non-disabling strokes.^86,87^ However, the premature termination of the PRISMS trial precludes its results from being considered definitive. The ARAMIS trial, conducted in China, subsequently provided compelling evidence, although further validation in other ethnic populations is required. Prespecified *post hoc* analysis of the ARAMIS trial found that in patients with mild non-disabling acute ischaemic stroke without large vessel occlusion, the proportion of bleeding events following intravenous rt-PA was higher than that following dual antiplatelet therapy, indicating intravenous rt-PA may be inferior to dual antiplatelet therapy in preventing early neurological deterioration and has worse safety profile.^88^ Two recent meta-analyses also support this finding.^89,90^ Thus, current guidelines do not recommend thrombolytic therapy for patients with minor non-disabling strokes. For patients with minor disabling deficits, secondary analysis of the AcT trial emphasizes that they typically exhibit significant occlusions on CT angiography,^91^ and would benefit from intravenous rt-PA. So, for these patients, intravenous thrombolysis is still recommended in various guidelines.

##### Influence of thrombus length

The length of the thrombus is also correlated with the effectiveness of intravenous thrombolysis treatment by rt-PA. Studies have shown that in acute middle cerebral artery strokes, if the thrombus length exceeds 8 mm, intravenous thrombolysis has little potential to recanalize the occluded vessel.^92^ It is also an independent predictor of early neurological deterioration in patients with minor stroke and large vessel occlusion following intravenous thrombolysis, and the longer the thrombus, the higher the risk of early neurological deterioration.^93^ For bridging therapy, early recanalization is crucial, and thrombus length is a strong independent predictor of failure to achieve early recanalization after intravenous thrombolysis, especially when the thrombus length exceeds 9 mm, the risk of the patient not achieving early recanalization significantly increases.^94^

### TNK-tPA

TNK-tPA, a representative of the new-generation thrombolytic agents, is being increasingly investigated for its efficacy and safety in acute ischaemic stroke patients (see Tables 2 and 3 and Supplementary Table 1). Its superior pharmacological properties have generated interest as a potential alternative to rt-PA. The most recent guidelines published in 2024 now equate the recommendation for rt-PA and TNK-tPA for intravenous thrombolysis within the first 4.5 h of onset.^5^ This development underscores the evolving role of TNK-tPA in stroke management, although further research is needed to fully establish its comparative efficacy and safety profile in different therapeutic strategies.

**Table 2:** Main published clinical trials on TNK-tPA (within 4.5 h time window)

Study name	Year	Country	Study design	Objective	Groups	Time window	Main results or conclusions
ATTEST	2015	UK	Phase 2, prospective, randomized, open-label, blinded end-point	Compare the efficacy and safety of TNK and rt-PA in a population without advanced imaging selection	0.25 mg/kg TNK-tPA 0.9 mg/kg rt-PA	4.5 h	There were no significant differences in efficacy and safety outcomes between the TNK group and the rt-PA group
NOR-TEST	2017	Norway	Phase 3, randomized, open-label, blinded endpoint, superiority	To investigate the safety and efficacy of TNK-tPA versus rt-PA in patients with AIS, most of whom had mild symptoms	0.4 mg/kg TNK-tPA 0.9 mg/kg rt-PA	4.5 h	TNK-tPA 0.4 mg/kg was not superior to rt-PA and showed similar safety results
NOR-TEST 2, part A	2022	Norway	Phase 3, randomized, open-label, blinded endpoint, non-inferiority	Establish the non-inferiority of TNK-tPA to rt-PA for patients with moderate or severe ischaemic stroke	0.4 mg/kg TNK-tPA 0.9 mg/kg rt-PA	4.5 h	TNK-tPA was inferior to rt-PA in terms of functional outcomes and safety. The trial was stopped early for safety reasons
TRACE	2021	China	Phase 2, multicentre, randomized, open-label, blinded-endpoint controlled study	Determine the safe dose range of TNK-tPA for Chinese patients with AIS	0.1 mg/kg TNK-tPA 0.25 mg/kg TNK-tPA 0.32 mg/kg TNK-tPA 0.9 mg/kg rt-PA	3 h	TNK-tPA was well tolerated in Chinese patients, with no significant difference in efficacy between the three groups of TNK-tPA and rt-PA
ACT	2022	Canada	Phase 3, multicentre, open-label, registry-linked, randomized, controlled, non-inferiority	Determine whether TNK-tPA will increase reperfusion compared to standard treatment	0.25 mg/kg TNK-tPA 0.9 mg/kg rt-PA	4.5 h	For primary outcomes, TNK-tPA was non-inferiority compared with rt-PA. They have similar safety. TNK-tPA is a reasonable alternative to rt-PA
TRACE-2	2023	China	Phase 3, multicentre, prospective, open-label, blinded-endpoint, randomized controlled	To test the hypothesis that TNK-tPA is non-inferior to rt-PA within 4.5 h	0.25 mg/kg TNK-tPA 0.9 mg/kg rt-PA	4.5 h	TNK-tPA is non-inferior to rt-PA in primary outcome, and they had similar safety, supporting the use of 0.25 mg/kg TNK-tPA as an alternative to the standard treatment
ATTEST 2	2024	UK	Phase 3, prospective, randomized, parallel-group, open-label	To investigate whether TNK-tPA was non-inferior or superior to rt-PA within 4.5 h of onset	0.25 mg/kg TNK-tPA 0.9 mg/kg rt-PA	4.5 h	TNK-tPA was non-inferior to rt-PA. TNK is easier to administer and should be preferred over rt-PA in the treatment of acute ischaemic stroke

rt-PA, Alteplase; TNK-tPA, Tenecteplase.

**Table 3:** Main published clinical trials on TNK-tPA (in the extended time window or bridging therapy)

Study name	Year	Country	Study design	Objective	Groups	Time window	Main results or conclusions
TAAIS	2012	Australia	Phase 2B, prospective, randomized, open-label, blinded endpoint	To explore whether TNK-tPA can achieve better reperfusion and recanalization rate in patients with stroke within 6 h.	0.1 mg/kg TNK-tPA 0.25 mg/kg TNK-tPA 0.9 mg/kg rt-PA	6 h	TNK-tPA was better than rt-PA in 24-h recanalization and clinical improvement, with 0.25 mg/kg being the most effective dose, and they had similar safety results
TEMPO-2	2024	International	Phase 3, multicentre, prospective, parallel-group, open-label, randomized controlled	To test the superiority of TNK-tPA over the standard non-thrombolytic therapy in patients with mild ischaemic stroke	0.25 mg/kg TNK-tPA Standard non-thrombolytic therapy	12 h	The two groups showed similar primary efficiency, but TNK-tPA had worse safety outcomes. TNK-tPA treatment for these patients may not be beneficial and could be harmful
TWIST	2022	International	Phase 3, investigator-initiated, multicentre, open-label, randomized	To determine whether TNK-tPA could improve functional outcomes within 4.5 h for wake-up stroke patients identified by non-contrast CT	0.25 mg/kg TNK-tPA + best standard treatment No TNK-tPA + best standard treatment	Wake-up ischaemic stroke within 4.5 h	TNK-tPA did not improve 90-day outcomes or safety outcomes compared to non-thrombolytic treatment and isn’t recommended for wake-up stroke patients selected by non-contrast CT
TIMELESS	2024	USA, Canada	Phase 3, multicentre, double-blind, randomized, placebo-controlled	To determine whether TNK-tPA is beneficial in patients who are imaged with perfusion imaging within 4.5–24 h of onset	0.25 mg/kg TNK-tPA Placebo	4.5–24 h	TNK-tPA did not result in better clinical outcomes than placebo. There were similar safety outcomes in both groups
TRACE 3	2024	China	Phase 3, multicentre, prospective, randomized, open-label, blinded endpoint	To determine the role of TNK-tPA in stroke patients with perfusion mismatch (without thrombectomy)	0.25 mg/kg TNK-tPA Standard medical treatment	4.5–24 h	In such patients, compared to standard drug treatment, TNK-tPA treatment can result in less disability and a similar survival rate
EXTEND-IA TNK	2018	Australia, New Zealand	Phase 2, multicentre, prospective, randomized, open-label, blinded-endpoint non-inferiority	To compare reperfusion and functional prognosis with the use of IV TNK-tPA before thrombectomy in contrast to rt-PA	0.25 mg/kg TNK-tPA 0.9 mg/kg rt-PA	4.5 h	TNK-tPA before thrombectomy is non-inferior to rt-PA in terms of reperfusion in the occluded areas, showing non-inferior functional outcomes, and has similar safety
EXTEND-IA TNK Part 2	2020	Australia, New Zealand	Phase 2, multicentre, prospective, randomized, open-label, blinded endpoint	To determine whether 0.4 mg/kg TNK-tPA can safely improve reperfusion before thrombectomy compared to 0.25 mg/kg of TNK-tPA	0.25 mg/kg TNK-tPA 0.4 mg/kg TNK-tPA	4.5 h	The 0.4 mg/kg TNK-tPA did not achieve more preoperative reperfusion than the 0.25 mg/kg TNK-tPA, and they had similar safety outcomes in these patients

rt-PA, Alteplase; TNK-tPA, Tenecteplase.

#### Optimizing TNK-tPA dosage

Initially, the optimal dose of TNK-tPA for stroke thrombolysis was uncertain. A pilot dose-escalation safety study found that doses ranging from 0.1 to 0.4 mg/kg were considered safe.^95^ However, the TNK-S2B study reported a higher risk of symptomatic intracerebral haemorrhage at 0.4 mg/kg (15.8%).^96^ Comparative analyses between 0.1 and 0.25 mg/kg TNK-tPA and 0.9 mg/kg rt-PA showed no significant differences in 90-day functional outcomes, leaving the optimal dose undetermined. The TAAIS study demonstrated better 24-h reperfusion rates and clinical improvement for TNK-tPA compared to rt-PA, with 0.25 mg/kg TNK-tPA showing the best performance,^97^ recommending it as a comparator dose despite the study’s small sample size (75 patients).

The TEMPO-1 study found that TNK-tPA at 0.25 mg/kg was more effective,^98^ consistent with the TAAIS study, and this dose was also used in the TEMPO-2 study.^99^ The NOR-TEST and NOR-TEST 2, part A trials compared 0.4 mg/kg TNK-tPA to rt-PA, with the former in minor strokes and the latter in moderate to severe strokes.^100,101^ While no significant differences were observed in NOR-TEST, the TNK-tPA group in NOR-TEST 2, part A experienced worse functional outcomes and safety, leading to early termination. The TRACE study conducted in East Asians found that Chinese patients tolerated 0.1, 0.25 and 0.32 mg/kg TNK-tPA well, with no difference in efficacy compared to standard rt-PA treatment.^102^ Collectively, these studies suggest that 0.25 mg/kg TNK-tPA may represent the optimal dose, offering the best efficacy without compromising safety (see Table 2).

#### Efficiency compared to rt-PA

Several studies have investigated whether TNK-tPA can serve as an alternative to rt-PA. The ATTEST trial, a phase II study, compared the efficacy of 0.25 mg/kg TNK-tPA and 0.9 mg/kg rt-PA in patients with stroke onset within 4.5 h and found no significant difference between the two groups.^103^ Subsequent large-scale trials, AcT and TRACE-2, provided compelling evidence that TNK-tPA at a dose of 0.25 mg/kg is non-inferior to rt-PA in terms of functional outcomes, quality of life and safety within 4.5 h of onset.^17,18^ The recently published ATTEST 2 study, the largest trial to date comparing TNK-tPA of 0.25 mg/kg to rt-PA of 0.9 mg/kg in acute ischaemic stroke, confirmed the non-inferiority of TNK-tPA at this dose compared with rt-PA, consist with AcT and TRACE-2 study.^104^ These studies demonstrated the efficacy of TNK-tPA in different ethnic populations. Additionally, shorter treatment time was associated with better clinical outcomes, emphasizing the importance of timely and efficient thrombolysis.^105^ The ease of TNK-tPA administration further supports the consideration of replacing standard rt-PA with 0.25 mg/kg TNK-tPA. A real-world, multicentre retrospective study demonstrates that TNK-tPA has comparable safety to rt-PA in thrombolytic therapy for acute ischaemic stroke (symptomatic intracerebral haemorrhage rate: 1.8 versus 1.98%) and may provide superior early neurological improvement and 90-day functional outcomes, supporting its broader application.^106^ Another real-world study indicates that TNK-tPA, as an alternative to rt-PA in stroke thrombolysis treatment, demonstrates significant advantages, including a higher likelihood of favourable outcomes, shorter treatment times and lower rates of symptomatic intracerebral haemorrhage.^107^ By comparing patient outcomes using the two drugs at different time periods within the New Zealand Central Region Stroke Network, this study provides compelling evidence of the efficacy of TNK-tPA in the real world and supports its effectiveness and safety as an alternative to rt-PA in stroke treatment. The most recent guidelines in China indeed recommend both TNK-tPA and rt-PA for intravenous thrombolysis within the 4.5-h window, highlighting the performance demonstrated by TNK-tPA.^5,108^ We are poised to enter an era of TNK-tPA therapy, potentially replacing rt-PA as a more beneficial treatment option for a broader patient population (see Table 2).

#### In extended time windows

The possibility of expanding the thrombolysis window for TNK-tPA was explored in the ROSE-TNK study. It demonstrated the safety and feasibility of TNK-tPA in treating stroke onset between 4.5 and 24 h, improving early neurological prognosis in patients with DWI/FLAIR mismatch, but found no significant differences in other outcomes, warranting confirmation from larger trials due to the small sample size.^109^ The TEMPO-1 study, focusing on minor ischaemic stroke with intracranial occlusion within 12 h, showed promising TNK-tPA results but lacked a non-thrombolysis control group and had a limited sample size.^98^ Subsequently, TEMPO-2, an international trial involving 886 patients, investigated the efficacy of 0.25 mg/kg TNK-tPA in minor ischaemic stroke and intracranial occlusion within 12 h. However, TNK-tPA did not yield benefits and might have been harmful compared to non-thrombolytic standard of care, leading to early study termination.^99^ Therefore, TNK-tPA is not recommended for routine intravenous thrombolysis in minor stroke with intracranial occlusion. Nevertheless, the ongoing TRACE IV study conducted by Yongjun Wang, compares TNK-tPA thrombolysis with dual antiplatelet therapy for minor stroke within 4.5 h, and its results will provide further evidence for TNK-tPA in thrombolysis of minor stroke.

For wake-up stroke within 4.5 h, the TWIST study screened patients for TNK-tPA thrombolysis within 4.5 h using non-contrast CT, but found no functional improvement at 90 days and increased symptomatic intracerebral haemorrhage ratio compared to non-thrombolysis.^110^ The sensitivity of non-contrast CT screening is lower than that of advanced imaging techniques, which may have resulted in the exclusion of patients with salvageable tissue during screening, thereby limiting the demonstration of therapeutic efficacy. Additionally, a higher proportion of patients in the control group underwent mechanical thrombectomy (14% in the control group versus 6% in the TNK-tPA group), which may have partially diluted the therapeutic effect of TNK-tPA. Delays caused by the COVID-19 pandemic and drug shortages may have further impacted the completeness of the study and the interpretation of its results. In addition, the publication of WAKE-UP and EXTEND IV studies during the study made it possible for patients to accept advanced imaging screening and choose rt-PA thrombolytic therapy, which affected the enrolment of patients who might benefit from TNK-tPA thrombolytic therapy, and there was a certain selection bias. Thus, this study did not successfully challenge the necessity of advanced imaging screening in reperfusion therapy for patients with wake-up stroke. The TIMELESS study, enrolling patients with onset times between 4.5 and 24 h, did not show superior clinical outcomes with TNK-tPA over placebo, although the 24-h complete recanalization rate in the TNK-tPA group was significantly higher than that in the control group, and patients with M1 segment occlusions tended to benefit from TNK-tPA thrombolysis.^111^ In the study, 77.3% of patients underwent mechanical thrombectomy, and the interval between thrombolytic administration and arterial puncture was relatively short (median time: 15 min). This short interval may have reduced the independent effect of intravenous thrombolysis, impacting its observation on the primary outcome. Additionally, the extension of the time window to 4.5–24 h may have diminished the efficacy of TNK-tPA, as the salvageable brain tissue potential decreases over time. Interestingly, the TRACE III determined the efficacy and safety of 0.25 mg/kg TNK-tPA thrombolysis in acute ischaemic stroke patients with large vessel occlusion and salvageable brain tissue identified on perfusion imaging within 4.5–24 h of onset (including wake-up and no witness stroke) and found that TNK-tPA thrombolysis was safe and significantly improved the prognosis.^112^ However, more compelling evidence is needed to verify the efficacy and safety of TNK-tPA beyond 4.5-h window. Ongoing trials such as EXIT-BT2 in China (Phase IV, evaluating TNK-tPA efficacy and safety within 4.5–6 h of acute ischaemic stroke onset) and RESILIENT (EXTEND-IV) in Brazil (Phase III, comparing TNK-tPA to placebo in non-large vessel occlusion ischaemic stroke within 4.5–12 h) are expected to update our understanding (see Table 3 and Supplementary Table 1).

#### Bridging therapy with TNK-tPA

Efforts have been made to employ TNK-tPA in bridging therapy for stroke. The EXTEND-IA TNK study demonstrated that 0.25 mg/kg TNK-tPA outperformed 0.9 mg/kg rt-PA in terms of faster and superior reperfusion in large vessel occlusion ischaemic stroke, positioning TNK-tPA as a viable alternative to rt-PA before endovascular therapy.^113^ The subsequent EXTEND-IA TNK Part 2 revealed no advantage in using 0.40 mg/kg TNK-tPA over 0.25 mg/kg for patients with planned endovascular thrombectomy for large vessel occlusion ischaemic stroke.^114^ Retrospectively studies showed that administration of TNK-tPA prior to mechanical thrombectomy was linked to higher early recanalization rates,^115^ and compared to rt-PA, the use of TNK-tPA in bridging therapy was associated with superior 3-month functional outcomes in patients with large core ischemia.^116^ Additionally, before mechanical thrombectomy, there was no significant difference in the probability of early recanalization between TNK-tPA and rt-PA. However, for thrombus larger than 10 mm, TNK-tPA is associated with a higher probability of early recanalization compared to rt-PA [OR 2.43 (95% CI, 1.02–5.81); *P* = 0.04].^115^ Due to the positive impact of TNK-tPA on bridging treatment, guidelines recommend considering 0.25 mg/kg TNK-tPA for bridging therapy in patients with large vessel occlusion suitable for mechanical thrombectomy prior to the procedure.^5-7,117^ (see Table 3).

Numerous international studies are currently investigating TNK-tPA in bridging therapy, such as BRIGE-TNK (China) and DIRECT-TNK (Brazil), which compare the efficacy of TNK-tPA within 4.5 h for bridging to endovascular thrombectomy in large vessel occlusion and anterior circulation strokes, respectively. TNK-PLUS and ATTENTION-IV LATE, both planned in China, will enrol patients with stroke onset between 4.5 and 24 h, comparing the efficacy of TNK-tPA combined with endovascular thrombectomy to standalone endovascular thrombectomy. POST-ETERNAL (Australia) compares the efficacy and safety of 0.25 mg/kg TNK-tPA thrombolysis ± mechanical thrombectomy in basilar artery occlusion within 24 h to current best practices, including rt-PA at 0.9 mg/kg or standard care/no thrombolysis ± mechanical thrombectomy. TRACE-V (China) compares the efficacy and safety of TNK-tPA thrombolysis ± endovascular thrombectomy in basilar artery occlusion within 24 h and intravenous rt-PA within 4.5 h from stroke onset or standard care (no thrombolysis) ± thrombectomy at treating clinician’s discretion. The outcomes of these trials remain unknown, and their results are eagerly awaited to potentially unveil credible treatment paradigms, offering benefits to a broader patient population (see Supplementary Table 1).

#### Intra-arterial thrombolysis with TNK-tPA

There are few research results on the feasibility of intra-arterial thrombolysis with TNK-tPA in conjunction with endovascular therapy. The BRETIS-TNK trial involved in the intra-arterial administration of TNK-tPA during endovascular treatment, showed a higher rate of reperfusion in the TNK-tPA group compared to the control group, though this represents only a preliminary conclusion.^118^ And the BRETIS-TNK II will be conducted to further verify the efficacy of intra-arterial TNK-tPA administration during endovascular thrombectomy. The ATTENTION IA trial investigates the efficacy and safety of intra-arterial administration of TNK-tPA following endovascular thrombectomy, but the results have not yet been disclosed. Further validation is required through the prospective, multicentre, randomized trials. Additionally, in the TECNO study, patients who have undergone incomplete mechanical thrombectomy will be treated with intra-arterial TNK-tPA or the best medical treatment, and the results are eagerly anticipated for their release (see Supplementary Table 1).

#### In minor stroke

The findings of the TEMPO-1 and TEMPO-2 studies provide significant insights into the safety and efficacy of TNK-tPA in the treatment of minor stroke. The TEMPO-1 study initially demonstrated the safety and feasibility of TNK-tPA,^98^ while its sample was only 50 and lacked a traditional control group, which undermines its reliability. The TEMPO-2 study, with a sample size of 886 patients, compared TNK-tPA intravenous thrombolysis with non-thrombolytic treatment,^99^ and found patients with minor strokes and intracranial occlusions do not benefit from TNK-tPA treatment, instead, TNK-tPA treatment increased the number of deaths [20 deaths (5%)] compared to the control group [5 deaths (1%); adjusted hazard ratio 3.8; 95% CI 1.4–10.2, *P* = 0.0085]. In addition, patients with vessel occlusion in the TNK-tPA group did not get better outcomes compared to the control group, though they exhibited higher recanalization rates. It is noted that medical care might improve the outcomes in patients with minor stroke, the subjects of TEMPO-2 trial, attenuating the benefits of thrombolysis. Most patients had relatively good collateral circulation, meaning supportive care alone could yield favourable outcomes. Meanwhile, because of the relatively mild neurological damage, the effects of vascular recanalization may not be adequately reflected in the current evaluation metrics.^99^ Furthermore, it is worth exploring whether ischemia-reperfusion injury could have adverse effects after vascular recanalization and following TNK-tPA treatment for minor stroke. The secondary analysis of the AcT trial suggested no differences in efficacy or safety between TNK-tPA and rt-PA in patients with minor stroke treated within 4.5 h of onset.^91^ Therefore, the current evidence remains limited, and the conclusions are inconclusive. Further randomized controlled trials are needed to evaluate the performance of TNK-tPA in minor stroke patients across different time windows.

#### Mobile stroke units and TNK-tPA

In mobile stroke units, intravenous thrombolysis has been a cornerstone in stroke management, traditionally relying on rt-PA. The question of whether TNK-tPA can match or even surpass rt-PA in mobile stroke unit was addressed by the TASTE-A study.^119^ The study concluded that TNK-tPA, compared to rt-PA, resulted in higher early reperfusion rates and did not report significant safety concerns. TNK-tPA demonstrated a faster initiation of intravenous thrombolysis in acute ischaemic stroke patients undergoing mobile stroke unit, further supporting its use, as recommended by the trial. Nonetheless, additional trials and case studies are necessary to consolidate and optimize the use and potential advantages of TNK-tPA in the management of mobile stroke units.

### Other new drugs

As a representative of third-generation thrombolytic agents, r-PA, derived from t-PA, offers affordability and a longer half-life. Ongoing investigations are exploring its efficacy and safety. A phase II, randomized, controlled trial in China aimed to establish the optimal safe dosage of r-PA for acute ischaemic stroke patients, enrolling 180 participants eligible for intravenous thrombolysis within 4.5 h of onset.^120^ Participants were randomly assigned to receive r-PA at doses of 12 + 12 mg or 18 + 18 mg via intravenous injection, or rt-PA at a dose of 0.9 mg/kg. The results demonstrated that r-PA was well tolerated and had comparable efficacy to rt-PA.

The phase III RAISE study, a prospective, open-label, non-inferiority, randomized trial conducted in 62 centres in China, compared the efficacy and safety of r-PA and rt-PA thrombolysis within 4.5 h of stroke onset.^8^ Recently disclosed results from the RAISE trial showed that 79.5% of patients in the r-PA group and 70.4% in the rt-PA group exhibited favourable functional outcomes (RR, 1.13; 95% CI, 1.05–1.21; *P* < 0.001 for non-inferiority and *P* = 0.002 for superiority). The RAISE study provides high-quality evidence supporting the use of r-PA for acute ischaemic stroke treatment, promising to influence future guidelines and offering clinicians and patients an economical and convenient thrombolytic option.

Non-immunogenic recombinant staphylokinase has been registered for acute ischaemic stroke thrombolysis in Russia. The administration involves dissolving 10 mg of the drug in 10 ml of 0.9% sodium chloride solution and delivering it via a single, 10-s intravenous push, with dosing independent of body weight, which simplifies drug administration. This recommendation is based on the FRIDA study, which showed that its efficacy in acute ischaemic stroke within the 4.5-h treatment window is non-inferior to rt-PA.^19^ Another observational study, REPIN, is currently recruiting to evaluate the efficacy and safety of non-immunogenic recombinant staphylokinase in routine clinical practice for acute ischaemic stroke reperfusion therapy. Further validation is required to establish its efficacy, safety and optimal treatment window.

## Safety of thrombolytics of different generations

The approval and adoption of a thrombolytic drug as a marketed therapy, apart from its efficacy, necessitates a thorough scrutiny of its safety and potential adverse effects. The benefits and risks must be carefully weighed. While some drugs may demonstrate promising efficacy, concerns over safety can pose challenges for their clinical trials and eventual approval. Intracerebral haemorrhage, extracranial bleeding and allergic reactions are among the safety concerns associated with thrombolytic treatments for acute ischaemic stroke. Despite the favourable pharmacological properties of these thrombolytics, they still carry a risk of bleeding or other adverse effects, impacting patient mortality or disability and thus their suitability.

### Streptokinase

Early clinical trials of streptokinase did not demonstrate a clear advantage over controls, with higher mortality and cerebral haemorrhage rates.^121^ The MAST-I trial observed a higher 10-day mortality rate in the streptokinase group (27 versus 12%, OR 2.7; 95% CI 1.7–4.3), along with systemic bleeding and allergic reactions, leading to trial discontinuation.^11^ Similar findings were seen in the MAST-E trial, with a higher 10-day mortality rate (34.0 versus 18.2%, *P* < 0.002) due to haemorrhagic transformation, particularly symptomatic intracerebral haemorrhage, resulting in early termination.^9^ The ASK trial showed higher mortality when treatment was delayed beyond 3 h (RR, 1.98; 95% CI, 1.18–3.35), while earlier treatment showed no such increase (RR, 1.11; 95% CI, 0.38–3.21). There were 4 cases of allergic shock and 58 instances (33.3%) of hypotension in the treatment group, but two other trials (MAST-I, MAST-E) did not report hypotension as a significant problem.^10^ Overall, streptokinase increases the risk of haemorrhagic transformation, mortality and other adverse effects, limiting its therapeutic benefits and suitability as a thrombolytic for acute ischaemic stroke.

### Urokinase

Regarding the safety of urokinase, an early study in monkeys found that increased risk of cerebral haemorrhage was associated with higher doses or prolonged urokinase administration.^122^ In clinical trials, 7% of acute ischaemic stroke patients experienced symptomatic intracerebral haemorrhage with local intra-arterial urokinase thrombolysis, with no clear association with common risk factors, such as hypertension, hypercholesterolemia, diabetes, baseline NIHSS score, treatment time, imaging findings or collateral circulation.^123^ Another trial reported a 27% mortality rate and some cases of intracerebral haemorrhage among patients treated with urokinase.^124^ An observational cohort study involving patients with large vessel occlusion stroke found no significant association between arterial urokinase administration and increased 90-day mortality or symptomatic intracerebral haemorrhage risk after unsuccessful or incomplete mechanical thrombectomy, suggesting the safety of urokinase during or after mechanical thrombectomy.^125^ However, these studies have limited sample sizes and primarily focus on local intra-arterial thrombolysis with urokinase.

In intravenous thrombolysis treatment, a Chinese retrospective study reported higher rates of extracranial bleeding in the high-dose urokinase group (1 200 000–1 500 000 IU) compared to the low-dose group (1 000 000 IU), with the low-dose group showing similar complication rates to rt-PA.^37^ The large-scale nationwide prospective Chinese registry study, INTRECIS, found no significant difference in symptomatic intracerebral haemorrhage, other bleedings, or mortality between intravenous urokinase and rt-PA within 4.5 h of stroke onset.^38^ Among the 1144 patients who received urokinase, 16 experienced symptomatic intracerebral haemorrhage (1.4%), 8 had other bleeding events (0.7%) and 58 died (5.1%). The study concluded that the safety and efficacy of urokinase are comparable to those of rt-PA, supporting its use in acute ischaemic stroke patients in China. However, further international, randomized controlled trials are needed to confirm these findings in other populations.

### rt-PA

As the most widely used thrombolytic agent for acute ischaemic stroke, rt-PA has been associated with increased adverse events despite its efficacy. The NINDS study reported higher symptomatic intracerebral haemorrhage rates (6.4%) within 36 h after the onset of stroke in patients treated with rt-PA compared to the placebo group (0.6%), although the mortality at 3 months was similar between the two groups.^4^ Subsequent studies, ATLANTIS and ECASS III, focused on patients with acute ischaemic stroke onset 3–4.5 h, and found increased intracerebral haemorrhage rates, including symptomatic intracerebral haemorrhage, in the rt-PA group, but no significant difference in mortality.^41,43^

The IST-3 study investigated rt-PA administration within 6 h and found higher rates of symptomatic intracerebral haemorrhage (7 versus 1%, aOR 6.94) and deaths (11 versus 7%, aOR 1.60) in the rt-PA group, as well as increased extracranial haemorrhage and allergic reactions.^45^ The EXTEND study further confirmed a higher risk of symptomatic intracerebral haemorrhage in the rt-PA group within the time window of 4.5–9 h.^47^ These findings suggest that the safety risks of rt-PA, particularly intracerebral haemorrhage, are heightened when administered outside the standard time window.

The ENCHANTED trial compared low-dose rt-PA (0.6 mg/kg) to the standard dose, revealing lower symptomatic intracerebral haemorrhage (1.0 versus 2.1%, *P* = 0.01) and 7-day mortality (0.5 versus 1.5%, *P* = 0.01) in the low-dose group, although non-inferiority of efficacy is yet to be established.^126^ A systematic review and meta-analysis observed that within the 4.5–9 h window or wake-up stroke, the rate of symptomatic intracerebral haemorrhage in the rt-PA group was higher compared to placebo, but this did not outweigh the overall net benefit of thrombolysis.^50^ Thus, while the risk of intracerebral haemorrhage and other adverse events increases with rt-PA administration beyond the standard time window, its efficacy remains a key factor for its continued use within a 4.5-h window.

The 2015 and 2018 AHA/ASA guidelines both stated that intravenous rt-PA in patients who have received a treatment dose of low molecular weight heparin within the previous 24 h is not recommended, otherwise the prophylactic doses, which were based on several studies with small sample sizes.^84,127^ A large observational study conducted on 1411 patients with acute ischaemic stroke, also confirmed that intravenous thrombolysis in patients with acute ischaemic stroke on treatment with prophylactic doses of low molecular weight heparin at stroke onset is not associated with an increased risk of symptomatic intracerebral haemorrhage or early death.^128^ However, more stronger evidences are needed from randomized controlled studies.

For acute ischaemic stroke patients who have recently taken oral anticoagulants, the safety of rt-PA intravenous thrombolysis is a problem that needs to be carefully concerned. At present, it is considered a contraindication for intravenous thrombolysis in patients with ischaemic stroke who have a recent intake of a direct oral anticoagulant in an international statemente,^84^ because of the increased risk of bleeding in this condition. However, two studies have shown that intravenous thrombolysis would not increase the safety risk of intracerebral haemorrhage for patients who have recently taken direct oral anticoagulants,^129,130^ and similar conclusions have been reached for patients who have recently taken non-vitamin K antagonist oral anticoagulants.^131,132^ In addition, a large retrospective cohort study assessed symptomatic intracerebral haemorrhage within 36 h after intravenous thrombolysis according to different selection strategies (direct oral anticoagulant-level measurements, direct oral anticoagulant reversal treatment, intravenous thrombolysis with neither direct oral anticoagulant-level measurement nor idarucizumab) and found that the unadjusted rate of symptomatic intracerebral haemorrhage was 2.5% (95% CI, 1.6–3.8) in patients taking direct oral anticoagulants compared with 4.1% (95% CI, 3.9–4.4) in control patients using no anticoagulants and after adjustment, recent use of direct oral anticoagulants (within 48 h) was not associated with increased risk of symptomatic intracerebral haemorrhage, regardless of whether patients were selected based on direct oral anticoagulant plasma levels, reversal with idarucizumab, or neither of those selection strategies.^130^ However, these are retrospective studies with certain limitations, and higher-level evidence is needed to further confirm these conclusions.

### TNK-tPA

Current research on TNK-tPA is gaining traction, potentially surpassing rt-PA due to its longer half-life and higher fibrin specificity (see Tables 2 and 3 and Supplementary Table 1). Numerous clinical trials are underway to validate its safety and efficacy. The magnitude of TNK-tPA dosage is pivotal in influencing therapeutic efficacy and safety. A pilot dose-escalation safety study found varying rates of asymptomatic intracerebral haemorrhage with different dosages (8% for 0.1 mg/kg, 32% for 0.2 mg/kg and 28% for 0.4 mg/kg), and recruitment for the 0.5 mg/kg group was halted due to symptomatic intracerebral haemorrhage cases.^95^ Subsequent studies, such as TNK-S2B, TAAIS and TEMPO-1, indicated better efficacy and comparable safety for 0.25 mg/kg TNK-tPA.^96-98^ What is more, in NOR-TEST, 0.4 mg/kg TNK-tPA for minor stroke had similar adverse event rates to rt-PA.^100^ The NOR-TEST 2, part A trial, which applied 0.4 mg/kg to moderate to severe strokes, showed inferior safety outcomes compared to rt-PA, leading to premature termination. Subsequently, the NOR-TEST 2, part B trial adjusted the dosage to 0.25 mg/kg,^101^ while the results are not yet revealed. Meanwhile, the TRACE trial demonstrated good tolerability of 0.1, 0.25 and 0.32 mg/kg in the Chinese population.^102^

The ATTEST, AcT and TRACE-2 studies compared 0.25 mg/kg TNK-tPA to the standard dose of rt-PA, finding similar safety outcomes.^17,18,103^ No significant differences were observed in short-term symptomatic intracerebral haemorrhage incidence or 90-day mortality. These studies indicate favourable tolerability of 0.25 mg/kg TNK-tPA across various populations.

Safety is also a critical evaluation of TNK-tPA treatment beyond the standard thrombolysis window. The ROSE-TNK trial reported three asymptomatic intracerebral haemorrhage cases without significant differences in adverse events between TNK-tPA and standard therapy, in acute ischaemic stroke patients with DWI/FLAIR mismatch with 4.5–24 h after onset.^109^ The TWIST and TIMELESS studies focused on awake stroke patients within 4.5 h of CT screening and patients with salvageable brain tissue in middle cerebral artery or internal carotid artery occlusion, respectively, showed similar safety profiles between TNK-tPA and control groups.^110,111^ TEMPO-1 confirmed the safety of TNK-tPA for minor stroke with intracranial occlusion within 12 h,^98^ while TEMPO-2 suggested potential harm with more deaths in the TNK-tPA group.^99^ The TRACE III study (4.5–24 h post-onset) reported a higher symptomatic intracerebral haemorrhage rate in the TNK-tPA group compared to standard treatment, while no difference in the 90-day mortality rate, systemic bleeding and severe adverse events.^112^ Further research, such as the TRACE IV study, may shed light on the safety of TNK-tPA for minor stroke beyond the thrombolysis window.

Currently, TNK-tPA has been approved for intravenous thrombolysis prior to thrombectomy. The CERTAIN study, enrolling 9238 patients, revealed lower symptomatic intracerebral haemorrhage rates with 0.25 mg/kg TNK-tPA compared to rt-PA, with consistent findings in thrombectomy and non-thrombectomy subgroups.^133^ The EXTEND IA TNK and Part 2 studies showed comparable safety profiles between TNK-tPA and rt-PA in bridging treatment.^113,114^ A retrospective analysis also confirmed the reliable safety of TNK-tPA in bridging treatment for large vessel occlusion stroke patients with extensive ischaemic cores when compared to rt-PA.^116^ The BRETIS-TNK trial suggested the safety of TNK-tPA for intra-arterial thrombolysis during endovascular therapy,^118^ but further large-scale trials are needed.

### Other drugs

For r-PA, the RAISE trial found 17 out of 700 patients (2.4%) experienced symptomatic intracerebral haemorrhage within 36 h post-onset in the r-PA group, without significant differences compared to the rt-PA group (2%, RR: 1.21; 95% CI, 0.54–2.75), though with higher intracerebral haemorrhage incidence and adverse events at 90 days.^8^ Non-immunogenic recombinant staphylokinase showed no significant differences in mortality, symptomatic intracerebral haemorrhage incidence or severe adverse events compared to rt-PA, suggesting comparable safety profiles.^19^ Further studies are needed to evaluate the safety of these two drugs for thrombolysis within or beyond 4.5 h.

## Clinical practices and future

Advancements in drug development have improved both the efficacy and safety of thrombolytics. From the first-generation agents like streptokinase, which lacked fibrin specificity and posed a risk of systemic bleeding, to the second-generation rt-PA with enhanced fibrin specificity, and now to third-generation drugs like TNK-tPA and r-PA, which offer extended half-life and improved thrombus penetration. These advancements have led to enhanced efficacy and safety.

### Administration methods

Both urokinase and rt-PA require prolonged intravenous infusion. Urokinase, for instance, is administered at a dose of 1 000 000–1 500 000 IU over 30 min. rt-PA is given intravenously at 0.9 mg/kg (up to 90 mg), with 10% of the dose injected in the first minute and the remainder infused over the next hour, due to its short half-life. In contrast, TNK-tPA can be administered as a 0.25 mg/kg intravenous push in 5–10 s, offering greater convenience.^5^ The RAISE study administered r-PA in two intravenous injections, 18 mg each, 30 min apart.^8^ Non-immunogenic recombinant staphylokinase can be administered as a single 10 mg intravenous injection, regardless of patient weight, simplifying the process further.^19^ Overall, administration methods have evolved to become more user-friendly, greatly facilitating thrombolytic therapy.

### Time is brain

The concept of ‘time is brain’ emphasizes the importance of rapid treatment to salvage the ischaemic penumbra. It is reported that for every 30-min reduction in onset-to-needle time, 2 out of 100 patients experience favourable functional outcomes.^105^ In Chinese populations, rt-PA showed shorter onset-to-treatment time (170 versus 190 min) and door-to-needle time (30 versus 35 min) compared to urokinase, facilitating earlier treatment initiation.^38^ Analysis of the AcT trial found that the type of thrombolytic drug (TNK-tPA versus rt-PA) did not alter the association between onset-to-needle time or door-to-needle time and primary clinical outcomes.^105^ However, the TNK-tPA real-world data of The New Zealand Central Region Stroke Network showed that patients treated with TNK-tPA had shorter door-to-needle time [median 52 (IQR 47–83) versus 61 (45–84) minutes, *P* < 0.0001] and needle to groin times [118 (74.5–218.5) versus 185 (118–255); *P* = 0.02)], compared to rt-PA.^107^ A retrospective study in New Zealand also yielded similar results, indicating that the median time for door-to-needle time (47 versus 48 min) or onset-to-needle time (129 versus 130 min) was comparable between the two groups.^134^ Another analysis of the New Zealand National Stroke Registry reported a shorter median door-to-needle time for TNK-tPA compared to rt-PA.^135^ A prospective observational study also linked the use of TNK-tPA to shorter door-to-needle time and door-in-door-out time in routine clinical practice.^136^ In China, compared with rt-PA, TNK-tPA could reduce significantly onset-to-treatment time (133 versus 163.72, *P* = 0.001) and door-to-needle time (36.5 versus 50, *P* < 0.001) in a retrospective study,^137^ and the incidence of door-to-needle time ≤45 min (65.83 versus 40.44%, *P* < 0.001) in the TNK-tPA group was significantly higher than in the rt-PA group. It is indicated that TNK-tPA may replace rt-PA in the future due to its efficiency in administration.

### Current recommendations

Currently, rt-PA remains the preferred thrombolytic for bridging therapy within 4.5 h, with a Class I, Level A recommendation, while TNK-tPA holds a Class II, Level B recommendation.^5^ For patients with anterior circulation large vessel occlusion suitable for endovascular treatment within 6 h, TNK-tPA may be considered for intravenous thrombolysis but not rt-PA, pending further randomized evidence (Class IIb, Level B).^117^ The efficacy and safety of TNK-tPA for bridging therapy with either intravenous or intra-arterial thrombolysis require additional validation.

### Cost considerations

TNK-tPA tends to have a lower median cost per visit compared to rt-PA (13 382 dollars versus 15 841 dollars, *P* < 0.001).^136^ Specific studies have focused on the cost-effectiveness of TNK-tPA and found that TNK-tPA is cost-effective compared with rt-PA in all scenarios (including acute ischaemic stroke patients with large vessel occlusion),^138^ or prior to thrombectomy.^139^ Previous studies were based on data from the developed countries. Similarly, in a low-income country like Nepal, the price of TNK-tPA (450 dollars) is nearly half that of rt-PA (1000 dollars),^140^ making it more accessible to impoverished populations. This more affordable cost allows patients from low-income groups to receive treatment with outcomes equivalent to those of rt-PA. r-PA, another affordable option, is also available.^120^ In China, where urokinase is approved, it may serve as a viable alternative for patients who cannot afford rt-PA.^37^ Similarly, in other low- and middle-income countries, urokinase also could be a cost-effective choice for intravenous thrombolysis.^141^

But, despite all this, the cost of intravenous thrombolysis in acute ischaemic stroke is relatively high, compared to traditional treatments, even with more significant benefits. More cost-effective, safe, and efficacious thrombolytic options are needed, especially for economically disadvantaged patients who may still be eligible for thrombolysis.

### Advanced neuroimaging and personalized treatment

The application of advanced neuroimaging technologies can refine the identification of eligible patients for thrombolysis, potentially widening the therapeutic window and improving outcomes. Acute ischaemic stroke patients exhibit diverse characteristics, necessitating personalized treatment strategies. Subgroup analysis of stroke patients can lead to tailored therapies, selecting the most appropriate treatment and deciding whether to proceed with thrombolytic therapy or conservative treatment for patients with atrial fibrillation, oral anticoagulants, renal impairment or advanced age, etc. More evidence would be obtained when more ongoing trials are completed, especially involved in TNK-tPA.

### Mobile stoke units

Furthermore, we are calling for the addition of mobile stroke units, which can facilitate early diagnosis and prompt treatment, shortening the time from symptom onset to treatment and optimizing thrombolysis.

### Future outlook

As more clinical trials and real-world data accumulate, thrombolysis for acute ischaemic stroke will have stronger evidence-based guidelines, offering physicians a wider range of options and improved treatment strategies. The future of thrombolysis for acute ischaemic stroke is promising, with a trajectory of improved treatments, thrombolytics development and technological advancements. These factors will contribute to better outcomes and increased hope for recovery for acute ischaemic stroke patients.

## Supplementary Material

fcaf164_Supplementary_Data

## Data Availability

Data sharing is not applicable to this article as no new data were created or analysed in this study.
