# Supplementary material for: Thrombolysis for acute ischaemic stroke: development and update
Source: Brain Commun. 2025 Apr 28;7(3):fcaf164. doi: 10.1093/braincomms/fcaf164 (PMC12053151; doi:10.1093/braincomms/fcaf164)
Supplement: fcaf164_Supplementary_Data [file fcaf164_supplementary_data.docx]

**Supplementary Table 1 Ongoing or unpublished clinical trials on TNK-tPA**

| **Key content** | **Study Name** | **ClinicalTrials.gov ID** | **Country** | **Study design** | **Groups** | **Time window** | **Study content** |
| --- | --- | --- | --- | --- | --- | --- | --- |
| Minor stroke | TRACE IV | NCT06414499 | China | Phase 3, multicenter, prospective, open-label, blinded-endpoint randomized controlled | a. 0.25 mg/kg TNK-tPA  b. standard medical care | 4.5 h | a. Recruiting 1874 patients with minor stroke (baseline NIHSS≤5) randomly assigned 1:1  b. Compare the short- or long-term efficacy and safety outcomes of the two therapies in the minor stroke. |
| Observational study | INTACT-China | NCT04588337 | China | Observational, prospective, multicenter, registry study | Intravenous TNK-tPA | 4.5 h | a. 1000 patients were recruited to undergo intravenous TNK-tPA  b. The efficacy and safety of TNK-tPA will be evaluated through the evaluation of long-term functional and safety outcomes |
|  | DISTAL-IVT | NCT05635786 | France | Observational | a. rt-PA (0.9 mg/kg)  b. TNK-tPA (0.25 mg/kg) | 4.5 h | 481 patients with distal vessel occlusion strokes undergo MRI within 1-2 hours of receiving different thrombolytics to compare the recanalization rate and clinical improvement of different thrombolytic therapies |
|  | TTT-AIS CHINA | NCT06078995 | China | Observational, multicenter, retrospective study | a. TNK-tPA  b. rt-PA | 4.5 h | 1200 patients will be included to compare the clinical efficacy and safety of two types of thrombolytics in the real world. |
|  | TETRIS | NCT05534360 | France | Multicenter ambispective observational study | TNK-tPA | Within 4.5 h or extended window | a. 5000 patients treated with TNK-tPA will be included in an observational study  b. Obtain data from routine clinical care to further determine the efficacy and safety of TNK-tPA in patients. |
| Dosage | ACT-WHEN-001 | NCT06320431 | Australia, Canada | Phase 3, prospective, randomized, controlled, open-label, parallel-group, blinded endpoint | a. TNK-tPA (0.25 mg/kg)  b. TNK-tPA (0.18 mg/kg)  c. No intravenous treatment | 4.5 h | a. 4000 patients will be recruited and randomized in different strata  b. Compare the efficacy and safety of different doses of TNK-tPA or non-intravenous thrombolysis in patients |
| In delayed time window | RESILIENT (EXTEND-IV) | NCT05199662 | Brazil | Phase 3, prospective, multicenter, randomized, controlled, double-blinded | a. Placebo  b. TNK-tPA (0.25 mg/kg) | 4.5-12 h | a. 360 patients with radiographically salvaging brain tissue will be randomly divided into two groups 1:1  b. Compare short-term or 90-day efficacy and safety assessment or longer follow-up. |
|  | EXIT-BT2 | NCT06010628 | China | Phase 4, prospective, randomized, open-label, blinded endpoint, multicenter | a. TNK-tPA 0.25mg/kg  b. standard stroke care based on national guideline | 4.5-6 h | a. 1250 patients will be recruited  b. Evaluate their short-term and long-term efficacy and safety after treatment |
|  | ETERNAL-LVO | NCT04454788 | Australia | Phase 3, prospective, randomized, open-label, blinded endpoint | a. TNK-tPA 0.25mg/kg  b. standard care (may include intravenous rt-PA) | 24 h | a. 740 patients were recruited and assigned to two groups  b. Efficacy outcomes and safety outcomes will be evaluated after treatment |
|  | TRACE-V | NCT06196320 | China | Phase 3, multicenter, prospective, open-label, blinded endpoint, randomized | a. TNK-tPA within 24 hours ± thrombectomy  b. rt-PA within 4.5 hours or standard care ± thrombectomy | 24 h | a. 452 patients will be randomly assigned to the two groups (1:1)  b. The efficacy and safety will be evaluated and compared after receiving different treatments |
|  | OPTION | NCT05752916 | China | Phase 4, multicenter, prospective, randomized, open-label, blinded-endpoint | a. TNK-tPA (0.25 mg/kg)  b. Antiplatelet agents | 4.5-24 h | a. 568 patients with non-large vessel occlusion (target mismatch on CT perfusion) will be recruited  b. The efficacy will be evaluated at different times by imaging or scales, and the safety will be also evaluated. |
|  | HOPE-BRIDGING | NCT05634382 | China | Phase 3 | a. thrombolysis agents (rt-PA or TNK-tPA) + thrombectomy  b. thrombectomy alone | 4.5-9 h | a. 222 patients were expected to be recruited and assigned to two groups  b. The efficacy and safety of the different treatments were evaluated and compared. |
| Intra-arterial thrombolysis | RESCUE-TNK | NCT05657470 | China | Phase 2/3, prospective, randomized, open-label, blinded endpoint, and multicenter trial | a. Intra-arterial TNK-tPA  b. Control group | 24 h | a. 80 patients will be randomly assigned to two groups 1:1  b. Evaluate the proportion of recanalization after treatment, short-term or long-term neurological outcomes and safety between the two groups. |
| Bridging therapy | BRIDGE-TNK | NCT04733742 | China | Phase 2/3, multicenter, randomized controlled Trial | a. TNK-tPA + thrombectomy  b. Thrombectomy alone | 4.5 h | a. 544 patients will be randomly assigned to the two groups  b. Evaluate short-term performance and 90-day functional outcomes to compare efficacy and safety. |
|  | DIRECT-TNK | NCT05199194 | Brazil | Phase 3, prospective, randomized, multicenter, double-blinded, placebo-controlled | a. Placebo + Thrombectomy  b. TNK-tPA (0.25 mg/kg) + thrombectomy | 4.5 h | a. 390 patients will be recruited and randomized 1:1 into two groups  b. Evaluate imaging or functional outcomes, and evaluate safety outcomes after treatment |
|  | 3T Stroke-III | NCT05745259 | China | Phase 3, prospective, randomized, open-label, blinded endpoint | a. 0.25 mg/kg TNK-tPA  b. 0.9 mg/kg rt-PA | 4.5 h before thrombectomy | a. 1630 stroke patients will be randomly assigned to two groups 1:1  b. Evaluate the short-term or long-term efficacy and safety of the two treatments. |
|  | TNK-CAT | NCT05626972 | Spain | Phase 3, multicenter, randomized, open-label blinded endpoint | a. 0.25 mg/kg TNK-tPA  b. 0.9 mg/k t-PA | 4.5 h before thrombectomy | a. 500 patients with suspected LVO will be randomly assigned 1:1 to two groups,  b. Different thrombolytic therapy will be followed by endovascular therapy  c. Efficacy and safety will be evaluated according to preoperative recanalization rate, neurological function outcome and other indicators. |
| Thrombectomy before intra-arterial thrombolysis | INSIST-IT | NCT05657457 | China | Phase 3, Prospective, randomized, open-label, blinded-end point, multicenter | a. Intra-arterial TNK-tPA  b. Control group | After thrombectomy | a. 228 patients will be recruited b. Evaluate short-term and long-term neurological outcomes and safety outcomes after treatment |
|  | ATTENTION-IA | NCT05684172 | China | Phase 2/3, multicenter, parallel-group, randomized clinical | a. Successful thrombectomy + intra-arterial TNK-tPA  b. Successful thrombectomy | After thrombectomy | a. 208 patients with large vessel occlusion of posterior circulation and successful thrombectomy will be recruited b. Evaluate the efficacy and safety in the short or long term after treatment after surgery |
|  | EXTEND-AGNES TNK | NCT05892510 | Australia | Phase 2b/3, multicenter, prospective, multi-arm multi-stage, seamless, randomized, placebo-controlled, double-blinded parallel | a. Thrombectomy + intra-arterial TNK-tPA  b. Thrombectomy + intra-arterial placebo | After thrombectomy | a. 462 patients with anterior circulation large vessel occlusion will be enrolled and randomly divided into two groups 1:1  b. Evaluate the short-term neurological improvement and long-term efficacy and safety outcomes by imaging or scale after treatment, and evaluate the performance of the therapy. |
|  | TECNO | NCT05499832 | Switzerland | Phase 3, multicenter, prospective, randomized, open-label, blinded endpoint (PROBE) proof-of-concept trial | a. Incomplete thrombectomy + intra-arterial TNK-tPA (3 mg)  b. Incomplete thrombectomy + standard of care | After thrombectomy | a. 156 patients who have incomplete reperfusion with residual occlusions after thrombectomy will be assigned to two groups  b. Evaluate the short- or long-term efficacy and safety. |
|  | BRETIS-TNK II | NCT05657444 | China | Phase 3, prospective, randomized, adaptive enrichment, open-label, blinded endpoint, multi-center study | a. Intra-arterial TNK-tPA during thrombectomy  b. Control group | During thrombectomy | a. 372 patients will be recruited into two groups  b. Compare short- or long-term efficacy and safety of the experimental group with the control group. |
|  | INSIST-TNK | NCT04201964 | China | Prospective, Single Arm, Pilot Study | Intra-arterial TNK-tPA (0.2-0.4 mg/min) immediately after thrombectomy device pass for 30-40 minutes | After insufficient recanalization thrombectomy | a. Enroll 30 patients  b. Assess the proportion of adequate recanalization after treatment, as well as short- or long-term functional outcomes and safety outcomes. |
|  | ARTERIAL-TNK-BAO | NCT05580822 | China | Phase 2, prospective, randomized, open-label, blinded endpoint, multicenter study | a. Intra-arterial TNK-tPA (0.4 mg/min)  b. Intra-arterial TNK-tPA (0.25 mg/min)  c. Intra-arterial placebo | After successful thrombectomy | a. 52 patients with acute basilar artery occlusion will be recruited  c. The efficacy and safety of the three treatments after thrombectomy will be judged. |
|  | ANGEL-TNK | NCT05624190 | China | Phase 4, multicenter, prospective, randomized, open-label, blinded end-point Trial | a. Thrombectomy + intra-arterial TNK-tPA  b. Thrombectomy + best medical management | After successful thrombectomy | a. 256 large vessel occlusion patients will be recruited  b. Compare neurological outcomes, as well as the short- or long-term efficacy and safety of TNK-tPA with the best medical management after treatment. |
| Bridging Therapy in the delayed time window | TNK-PLUS | NCT06221371 | China | Phase 3, multi-center, prospective, open-label, blinded endpoint, randomized controlled | a. Direct thrombectomy  b. 0.25 mg/kg TNK-tPA + thrombectomy | 4.5-24 h | a. 390 patients will be randomly divided into two groups according to 1:1  b. The 90 days mRS or short-term vascular recanalization will be evaluated, and the efficacy and safety will be compared. |
|  | ATTENTION-IV LATE | NCT05701956 | China | Phase 3, multicenter, prospective, controlled clinical trial with open-label treatment and blind outcome assessment | a. TNK-tPA (0.25 mg/kg) + thrombectomy  b. Thrombectomy alone | 4.5-24 h | a. 332 patients within 4.5 to 24 hours of onset will be recruited  b. Compare postoperative imaging results, as well as short- or long-term neurological outcomes and safety outcomes |
|  | POST-ETERNAL | NCT05105633 | Australia | Phase 2b/3, multi-arm/ multi-stage, multiregional, multicenter, prospective, randomized, open-label, blinded endpoint | a. TNK-tPA (0.25 mg/kg) +/- thrombectomy  b. Standard of care (rt-PA 0.9mg/kg or no lysis) +/- thrombectomy | 24 h | a. 688 patients of basilar artery occlusion will be randomly divided into two groups.  b. The efficacy and safety of TNK-tPA (0.25 mg/kg) +/- thrombectomy will be evaluated and compared with the standard therapy +/- mechanical thrombectomy |

rt-PA, Alteplase; TNK-tPA, Tenecteplase.

**Supplementary Reference:**

ClinicalTrials.gov. <https://www.clinicaltrials.gov/>. Accessed 21 April 2025.
